# Enhancing the Antioxidant Properties of Carrot Wastes Through Lactic Acid Fermentation and Thermophysical Treatments

**DOI:** 10.3390/foods15060985

**Published:** 2026-03-10

**Authors:** Claudia Bas-Bellver, Cristina Barrera, Lucía Seguí

**Affiliations:** Instituto Universitario de Ingeniería de Alimentos—FoodUPV, Universitat Politècnica de València, Camino de Vera, s/n, 46022 Valencia, Spain; clbabel@etsiamn.upv.es (C.B.-B.); mcbarpu@tal.upv.es (C.B.)

**Keywords:** carrot waste LAB fermentation, ultrasounds, microwaves, antioxidants, microstructure

## Abstract

Carrot wastes contain valuable bioactive compounds, particularly carotenoids, phenolics, and dietary fibre, whose concentration and bioaccessibility can be enhanced through structural and biochemical modifications induced by thermophysical and biological treatments, providing valorization potential. This study evaluated the effects of microwave treatment (1.5 or 3 W/g for 2, 4, and 6 min), ultrasound treatment (100 or 200 g at 40 kHz for 5, 10, or 15 min), and lactic acid fermentation with *Lactiplantibacillus plantarum*, *Ligilactobacillus salivarius*, and *Limosilactobacillus reuteri* for 48 h on the plant cell structure and physicochemical and antioxidant properties of fresh-cut carrot wastes. Among the tested strains, *L. salivarius* showed the highest growth after 24 h of fermentation (from 8.8 to 9.7 log_10_ CFU/g), increasing total phenolic content by 1.3-fold. Antioxidant activity improved, with DPPH peaking at 48 h (2.7-fold) and ABTS increasing from early fermentation stages. Ultrasounds enhanced antioxidant properties, so that 100 g–15 min led to maximum increases in total phenols, flavonoids, and ABTS (fold-increase of 1.3, 2.7, and 1.4, respectively), while DPPH values peaked after 5 min (70% increase). Microwave treatment at 3 W/g increased total phenols and flavonoids 1.3-fold, although prolonged exposure reduced antioxidant activity. Overall, selected thermophysical and fermentation treatments may enhance the bioactive potential of carrot wastes.

## 1. Introduction

Carrot (*Daucus carota* L.) is one of the most popular root crops in the world and one of the 10 most economically valued crops [1]. It is consumed both fresh and processed in many forms, such as juices, beverages, sweets, canned or dehydrated. Carrots vary widely in size, shape and colour, with orange, purple, red, white and yellow varieties [2]. Orange-rooted carrots cultivars are rich in antioxidants like carotenoids (mainly β-carotene and α-carotene) [3] and polyphenols (mainly hydroxycinnamic acid derivatives) [4,5]. The consumption of carrots and carrot-derived products has grown rapidly in recent years due to its recognition as a natural source of antioxidants and bioactive compounds that provide health benefits [6], reaching a global production of 43 million tons in 2024 [7]. Consequently, the amount of carrot waste generated annually has also increased significantly. Carrot residues are produced both during industrial processing (for juice, canning and other industries) and in the early stages of manufacturing. During the latter, carrot discards are generated after harvesting due to high quality standards, and minimally processing lines such as fresh-cut and frozen vegetables ones, generate remarkable amounts of clean and nutritious carrot wastes.

Carrot residues may account for up to 50% of the raw material processed; this biomass has traditionally been used for animal feed or fertilizer, or disposed of in landfills [8]. However, these residues contain bioactive compounds that may have a second use in the food, biotechnology and pharmaceutical industries, among others [9]. The sustainability and circularity approach to new food designs aims to minimize food waste and to valorize the residues by reintroducing them into the food chain as nutritious ingredients. This aligns with the Sustainable Development Goals (SDGs) [10], mainly SDG12 “Responsible production and consumption” and SDG3 “Health and well-being”. In this context, the transformation of carrot residues into stable powders has emerged as a promising strategy, as it enables the integral valorization of this biomasssand facilitates its incorporation as functional ingredients in food formulations [11,12]. Alternatively, carrot residues may be used as a raw material for the extraction of specific bioactive compounds intended for food, nutraceutical, cosmetic, or pharmaceutical applications [10]. While extraction-based approaches allow for the selective recovery of high-value compounds, powder production offers the advantage of whole-residue valorization, minimizing waste generation and processing losses. In both strategies, pretreatment technologies play a key role in determining the accessibility, stability, and functionality of bioactive compounds.

Pretreatments may involve biological or thermophysical approaches, with each inducing specific modifications to the plant’s cellular structure, composition, and bioactive profile, which ultimately influence the physicochemical characteristics and antioxidant properties. Fermentation with lactic acid bacteria (LAB) is a traditional and energy-efficient preservation method that may improve food properties by increasing bioactive compounds’ release or transformation into more active forms by microbia [13,14]. Hydrolytic and oxidative enzymes secreted by LAB are responsible for both the release and biotransformation of phenolics. Hence, this process improves organoleptic properties, digestibility, and nutrient bioavailability [15]. Among the thermophysical pretreatments, microwave and ultrasound treatments have gained increasing attention due to their ability to modify plant tissue structure through distinct physical mechanisms. Microwave processing involves the interaction between electromagnetic waves and water molecules and ions, generating rapid heating and localized hotspots which lead to the fast vaporization of trapped water, thus disrupting cell walls and membranes [16,17,18]. This breakage of the cellular matrix may release compounds of interest that were previously contained in cells and vacuoles or bound to complex polysaccharides. Ultrasonication is characterized by promoting cavitation phenomena [19], causing changes in the plant tissue, such as pores in cell membranes, cell disruption, enzyme denaturation or microscopic channel formation [20], and facilitating the release of antioxidant compounds and the diffusivity of water within the plant material [21]. Therefore, ultrasounds are expected to promote the disruption of the cellular structure and contribute to the release of antioxidant compounds, such as phenolic constituents.

However, the effectiveness of these strategies as pretreatments is highly dependent on the processing conditions applied, and the inadequate selection of treatment parameters may not only limit their benefits but could also adversely affect the content, stability, and release of bioactive compounds. Significant efforts have been devoted to the study of using microwaves and ultrasounds in drying operations when applied either simultaneously or prior to dehydration and to assist the extraction of bioactive compounds from cellular matrices [12], but there is still little knowledge about their impact as pretreatments for obtaining functional food ingredients from agro-industrial residues. Regarding fermentation, the growing interest in this biotransformation process has shifted the focus toward its use as a waste valorization strategy. Although some of these technologies may have been individually studied in plant matrices, comparative evaluations under controlled conditions are scarce, particularly for fresh-cut carrot discards. A systematic comparison is therefore necessary to identify optimal strategies for maximizing bioactive potential.

In this context, the aim of this work was to investigate the effect of different thermophysical (microwave and ultrasonication) and biological (lactic-acid fermentation) treatments on the physicochemical and antioxidant properties, as well as on the cell structural integrity, of fresh-cut carrot discards.

## 2. Materials and Methods

### 2.1. Raw Material

Carrot (*Daucus carota*, L.) waste was sourced from a ready-to-eat carrot sticks line (IV-range) supplied by Agrícola Villena, S.L. (Alicante, Spain). Carrot discards consisted of the sticks and portions remaining after processing, obtained from carrots that had already been washed, peeled, and cut into ready-to-eat sticks. The main reasons for discarding this material were its irregular shape, inadequate size, or the presence of peel or blemishes. When received, carrot wastes were disrupted in a Thermomix^®^ food processor (Vorwerk, Madrid, Spain) at 10,000 rpm for 10 s to obtain pieces of about 5 mm [22], before they underwent the processes described below. This material was used as the control and named disrupted carrot waste.

### 2.2. Biological and Thermophysical Treatments of Carrot Wastes

#### 2.2.1. Fermentation Trials

*Lactiplantibacillus plantarum* CECT 749, *Ligilactobacillus salivarius* CECT 4063 and *Limosilactobacillus reuteri* CECT 925 (Colección Española de Cultivos Tipo, Valencia, Spain) were used in the fermentation trials. The selection of these strains was based on their documented probiotic potential, their recognition as safe for food applications, and their use in previous studies, demonstrating their ability to adapt and ferment plant-based matrices.

In all cases, recovery of the strain preserved in cryo-vials was carried out in Man, Rogosa and Sharpe (MRS) broth (SharlauChemie^®^, Barcelona, Spain) and kept at 37 °C for 24 h in an incubation oven (Incugidit, PSelecta, Barcelona, Spain), as described in [23], to obtain a starter inoculum containing 10^8^–10^9^ CFU/mL (measured by plate count). For fermentation assays, 200 g of the disrupted carrot waste was placed in sterile glass jars with twist-off lids and pasteurized at 72 °C for 1 min in a hot water bath (Precisterm, PSelecta, Barcelona, Spain). The initial microbial load was therefore reduced to favour the growth of the inoculated microorganism. Pasteurized samples were left to cool down to room temperature, inoculated with 2 mL of the prepared inoculum and incubated at 37 °C. After 12, 24 and 48 h of fermentation, pH was measured, and plate-counting following plate-seeding was performed to determine the microbial content of the fermented residue.

#### 2.2.2. Ultrasound Treatment Trials

Disrupted carrot discards were distributed into 250 mL twist-off glass bottles containing 100 or 200 g of the disrupted carrot waste and immersed in an ultrasound bath (Ultrasons-H, Selecta, Barcelona, Spain), which operates at a fixed frequency of 40 kHz. For a given energy input and treatment duration, the energy density received by the sample depends on the sample’s mass. Therefore, two different sample masses were proposed to assess the effectiveness of the US treatment at two different energy densities. Ultrasound was applied at 5, 10 or 15 min.

#### 2.2.3. Microwave Treatment Trials

Disrupted carrot waste was subjected to microwave treatment in 250 mL twist-off glass jars containing 100 g of carrot waste. Samples were individually placed in a domestic microwave oven equipped with a rotating plate (Samsung GW72N, Samsung Electronics, Suwon, Republic of Korea), and power was set to 1.5 or 3 W/g for 2, 4 and 6 min. These powers were selected based on the group’s previous experience in assessing the impact of microwaves on other vegetable matrices [17,24].

### 2.3. Analytical Determinations

The analytical determinations described below were performed to characterize the carrot residue, both after disruption (control) and after being processed, according to the treatments and conditions described above.

#### 2.3.1. Physicochemical and Antioxidant Properties

**Water activity** (a_w_) was determined at 25 °C using a dew point hygrometer (Aqualab 4TE; Decagon devices Inc., Pullman WA, USA). **Moisture content** (x_w_) was assessed gravimetrically through the AOAC method (AOAC 934.06) [25], by drying until constant weight in a vacuum oven (VacioTemp-T Selecta, Barcelona, Spain) (P = 10 mm Hg) at 60 °C. **Total soluble solids content** (x_ss_) was calculated from the Brix values measured at 20 °C with a thermostatic Abbe refractometer (model NAR-3T, Atago, Tokyo, Japan), according to the ISO 1743:1982 method [26], in the liquid phase, which was obtained by pressing the samples (both fresh and treated).

To determine the antioxidant properties, extracts were prepared by combining 4 g of the sample with 10 mL of an 80% (*v*/*v*) methanol:water solution (Scharlab S.L., Barcelona, Spain). The mixture was shaken for 1 h on a horizontal shaker (COMECTA WY-100, Barcelona, Spain) at 200 rpm and subsequently centrifuged at 10,000 rpm for 5 min using an Eppendorf Centrifuge 5804/5804R (Eppendorf SE, Hamburg, Germany). The supernatants were collected for further analysis. **Total phenolic content** was quantified by the Folin–Ciocalteu spectrophotometric method [27,28]. Briefly, 0.125 mL of extract, 0.125 mL of Folin–Ciocalteu reagent (Scharlab S.L., Barcelona, Spain) and 0.5 mL of bidistilled water were combined in a cuvette. After 6 min of reaction in darkness, 1.25 mL of 7% (w/v) sodium carbonate solution and 1 mL of bidistilled water were added. Absorbance was measured at 760 nm after 90 min in darkness, and results were expressed as mg of Gallic Acid Equivalents (purity of gallic acid ≥ 98%, Sigma-Aldrich, St Louis, MO, USA) per g of dry matter (mg GAE/g_dm_). **Total flavonoid content** was measured using the modified aluminum chloride (AlCl_3_) colorimetric assay [29]. For this, 1.5 mL of the extract was mixed with 1.5 mL of a 2% (w/v) AlCl_3_ solution (Thermo Fisher Scientific Inc., Waltham, MA, USA) in methanol. After 10 min in darkness, absorbance was measured at 368 nm and the results were expressed as mg of Quercetin Equivalents (purity of quercetin ≥ 95%, Sigma-Aldrich, St Louis, MO, USA) per g of dry matter (mg QE/g_dm_). **Antioxidant activity** was evaluated by the DPPH (1,1 diphenyl-2-picryl hy- drazyl) and ABTS (2,20-azobis-3-ethyl benzothiazolin-6-sulphonic acid) radical assays. For the DPPH assays [30,31], 0.1 mL of extract was added to 2.9 mL of a 0.06 mM DPPH solution (Thermo Fisher Scientific Inc., Waltham, MA, USA) in methanol and incubated in darkness for 60 min before measuring the absorbance at 515 nm. For the ABTS assay [32], 0.1 mL of extract was mixed with 2.9 mL of ABTS+ (VWR International LLC, Radnor, PA, USA) solution in phosphate buffer with an absorbance of 0.70 ± 0.02 at 734 nm and allowed to react for 7 min prior to absorbance measurement at 734 nm. The results in both the DPPH and ABTS assays were expressed as mg of Trolox Equivalent (purity ≥ 97%; Sigma-Aldrich, St Louis, MO, USA) per g of dry matter (mg TE/g_dm_). All absorbance readings described in this section were performed using a Cary 60 UV/Vis spectrophotometer (Agilent Technologies, Santa Clara, CA, USA).

#### 2.3.2. Evolution of Microbial Counting and pH During Fermentation

In fermented samples, microbial counting was performed and pH was determined at 0, 12, 24 and 48 h of fermentation. Microbial counts were carried out by the serial dilution method in sterile buffered peptone water (Scharlab, Barcelona, Spain) with subsequent plate-seeding. For solid samples, initial dilution was prepared by mixing a 3 g sample and 27 mL of sterile buffered peptone water (ScharlauChemie^®^, Barcelona, Spain) in a sterile stomacher bag and homogenizing for 2 min using a stomacher (Interscience, BagMixer^®^ 400 model, St Nom, France). Subsequent tenfold serial dilutions up to 10^−8^ g/L) were prepared, and appropriate aliquots were plated onto MRS agar (ScharlauChemie^®^, Barcelona, Spain). After incubation at 37 °C for 24–48 h, the resulting colonies were enumerated. pH was measured with a digital pHmeter S20 SevenEasyTM (Mettler-Toledo Inlab, Barcelona, Spain).

#### 2.3.3. Cryo-Field Emission Scanning Electron Microscopy (Cryo-FESEM)

The microstructural changes induced by lactic acid fermentation and thermophysical treatments were evaluated using Cryo-FESEM Observations were conducted at the Servicio de Microscopía of the Universitat Politècnica de València, utilizing a Zeiss Ultra 55 microscope (Carl Zeiss Microscopy GmbH, Oberkochen, Germany) equipped with a Quorum PP3010 sublimation chamber. Samples were affixed to the analytical plate using a cement composed of colloidal graphite in water (G303 Colloidal Graphite AQUADAC, Oxford Instruments plc, Abingdon, Oxfordshire, England) combined with a tissue fixative (Tissue-Tek AutoTEC^®^ a120, Sakura Finetek USA, Inc., Torrance, CA, USA). Conductive strings of cement were created to enhance the distribution of the electron flux.

Vegetal tissue was freeze-dried under vacuum using liquid nitrogen (−196 °C) prior to fracturing and drying in the sublimation chamber at −90 °C and 10^−7^ mbar for 15 min. Platinum sputtering was performed at 5 mA for 20 s. Finally, samples were observed at −150 °C using the ZEISS SmartSEM software (version 5.06 with Service Pack 4), with an acceleration voltage of 10–20 kV.

### 2.4. Statistical Analysis

To minimize uncertainties arising from raw material variability, a different batch was assigned to each treatment (ultrasound, microwave, and fermentation). Therefore, differences observed within a specific treatment were attributed to the different conditions applied, whereas differences in control samples among the different treatments assayed were attributed to batch-to-batch variability. All analytical determinations were performed in duplicate on two or three independent samples (experiments), as indicated in each case.

Data were statistically analyzed using Statgraphics Centurion XIX software (Statpoint Technologies, Inc., Warrenton, VA, USA). Analyses of variance (one-way ANOVA and multifactorial ANOVA) were carried out with a confidence level of 95% (*p*-value < 0.05). When the ANOVA analysis was significant, Fisher’s LSD method was used for mean comparison. Assumptions of normality and homogeneity of variance were verified prior to analysis. All analyses were performed at least in triplicate.Values in tables are presented as mean ± standard deviation, using significant figures according to the theory of error. 

## 3. Results and Discussion

### 3.1. Impact of Fermentation with Lactiplantibacillus plantarum, Limosilactobacillus reuteri and Lactobacillus salivarius on the Physicochemical and Antioxidant Properties of Carrot Residues

**Figure 1:**
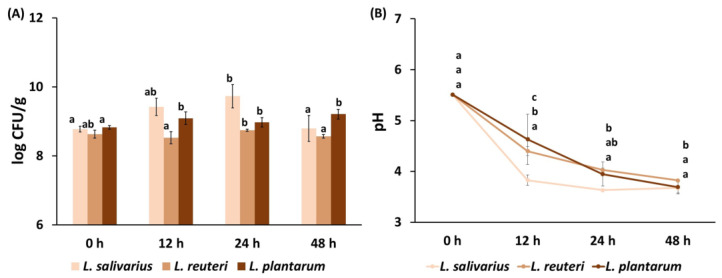
(**A**) Concentration (Log CFU/g) of *Lactiplantibacillus plantarum CECT 749*, *Ligilactobacillus salivarius CECT 4063* and *Limosilactobacillus reuteri* and (**B**) pH in pasteurized carrot residue before (0 h) and after 12, 24 and 48 h of fermentation. Error bars represent the standard deviation of two independent samples (*n* = 2), measured in duplicate. ^a,b,c^ different letters in the same series indicate statistically significant differences at a 95% confidence level.

Figure 1 shows the bacterial concentration (log CFU/g product) obtained for the three strains tested at 12, 24 and 48 h of fermentation, as well as the pH evolution during the fermentation of pasteurized disrupted carrot waste with the following potential probiotic strains: *Lactiplantibacillus plantarum*, *Limosilactobacillus reuteri* and *Lactobacillus salivarius*. During fermentation, the highest counts were obtained for *L. salivarius*, with a maximum around 10^9^ CFU/g after 24 h, which coincided with the lowest pH value registered, indicating higher fermentative performance and the better adaptation of this microorganism to the carrot matrix. Achieving high viable counts at the end of fermentation is particularly relevant, as losses in cell viability are expected during further processing and storage prior to use. After 24 h, the number of viable cells of *L. salivarius* started to decrease and the pH remained constant, as observed for *L. reuteri*. Other authors reported a decrease in lactic acid bacteria after showing maximum growth at 24 h when fermenting fruit beverages [33]. In contrast, *L. plantarum’s* maximum growth was reached at 12 h and remained constant until 48 h, while pH continued to decrease. In all cases, the viable count was found to be higher than 10^7^ CFU/g, so all the resulting products could potentially be considered probiotic, as approximately 10^6^–10^8^ CFU/g of viable cells is required for a product to be designated probiotic [34,35].

Based on these results, *L. salivarius* was selected for further assays, and new fermentation trials were conducted to evaluate changes in the physicochemical and antioxidant properties of the plant material during fermentation. Table 1 shows the values obtained for the moisture content (%), water activity (a_w_) and soluble solids content (x_ss_) of carrot residues throughout fermentation with *L. salivarius*.

Fermentation did not imply significant changes in the moisture content and water activity of the residues (*p*-value ≥ 0.05), while there was a significant decrease in the soluble solids content as fermentation progressed (*p*-value < 0.05). Other studies also observed a decrease in soluble solids during the fermentation of different vegetables, including tomato, red chilli, pumpkin and carrot, with the latter showing the greatest decrease in soluble solids content [36]. This result can be attributed to the bacterial consumption of sugars during fermentation; nevertheless, it should be noted that microorganisms may not only consume the simple sugars that are initially present in the vegetable matrix, but also sugars released from complex polysaccharides due to enzymatic action, so the observed changes in soluble solids may underestimate the total amount of carbohydrates metabolized. A progressive decrease in pH is observed as fermentation progresses, reaching a minimum after 24 h, with no more significant changes at 48 h. The production of organic acids during fermentation, such as lactic acid, reduces the pH of the medium [37,38]. Notably, the fermented samples reached a pH below 4.6, a value widely recognized as a critical threshold for food safety [39].

**Figure 2:**
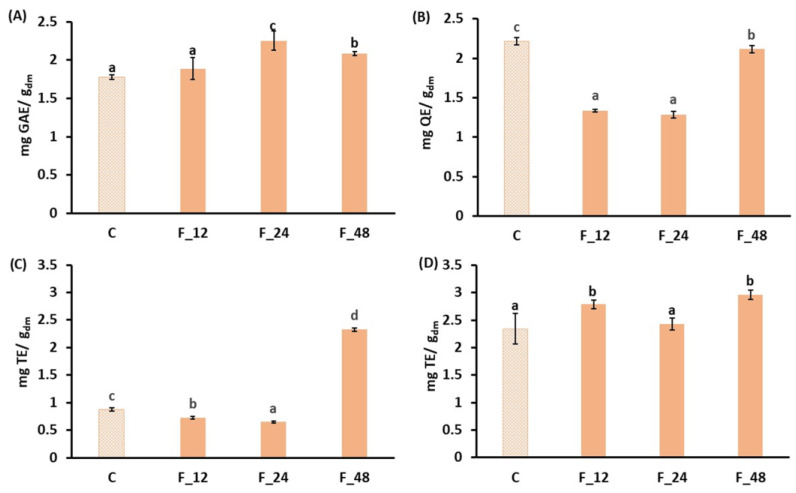
Evolution of the antioxidant properties of carrot residue during fermentation with *L. salivarius*. (**A**) Total phenols content (mg GAE/g_dm_); (**B**) total flavonoid content (mg QE/g_dm_); (**C**) DPPH antioxidant capacity (mg TE/g_dm_); (**D**) ABTS antioxidant capacity (mg TE/g_dm_). C: control sample (disrupted and pasteurized carrot residue); F_12, F_24 and F_48: carrot residue fermented with *L. salivarius* for 12, 24 and 48 h, respectively. Error bars represent the standard deviation of two independent samples (*n* = 2), measured in duplicate. ^a,b,c,d^ different letters for the same compound indicate statistically significant differences at the 95% confidence level.

The antioxidant properties (total phenols, total flavonoids and antiradical activity) of carrot waste during fermentation with *L. salivarius* are summarized in Figure 2. Fermentation can lead to the release of bound phenolics and their biotransformation, which ultimately influences the bioactivity and bioavailability of the resulting products [40]. Notably, LAB secrete a series of hydrolytic and oxidative enzymes, including esterases, decarboxylases and phenolic acid reductases, which are responsible for the release and biotransformation of phenolic acids. Total phenols increased significantly during fermentation (Figure 2A), particularly after 24 h, when the values were 1.3 times higher than those in the unfermented residue. During carrot waste fermentation by LAB, the antioxidant properties of the product increase when carbohydrates are consumed for carbon and nitrogen, breaking down the plant cell wall structure [41]. Other studies on LAB-fermented carrot products also reported that the LAB enzymatic metabolism degrades complex polyphenols into simpler compounds, thus releasing free phenolics, which increase total phenolic content [42,43]. However, at 48 h, a decrease in phenolic content is observed, suggesting that lactic acid bacteria could degrade phenols to continue growing [44,45]. Flavonoids exhibited a significant decrease (*p*-value < 0.05) during fermentation compared to the control (Figure 2B), although this partly recovered after 48 h of fermentation. This suggests a possible degradation of flavonoids caused by *L. salivarius*, or their conversion into less AlCl_3_-reactive forms [44]. Various lactic acid bacteria strains demonstrated different abilities to biotransform flavonoids. Gao et al. [46] reported that LAB fermentation may have a variable impact on flavonoid content depending on the microorganism and strain used. A targeted metabolomic analysis performed by these authors revealed that high-molecular-weight flavonoids, such as rutin, epicatechin, epigallocatechin and others, were more abundant in raw materials than in fermented samples, whereas the upstream components of the synthesis pathway, which were naringenin chalcone and eriodictyol, were scarce in the raw materials but abundant in fermented products. In addition to the changes in the relative content of different flavonoid compounds, their different reactivities with aluminum chloride may also influence the total flavonoids. Other authors [47] observed a decrease in total flavonoid content after 24 h of LAB fermentation of fig juice, but reported that other authors confirmed a further increase when extending fermentation to 48–72 h.

The antioxidant capacity also evolved during fermentation (Figure 2C,D), achieving different results when DPPH or ABTS radicals were used for the test. According to the DPPH radical method, the antioxidant capacity decreased by 17% during the first 24 h of fermentation, but after 48 h the result was 2.7 times higher than the control. In contrast, ABTS antioxidant capacity remained constant or increased during fermentation, reaching the highest value at 48 h, which was 1.3 times higher than that of the unfermented carrot residue. The increase in antioxidant activity during fermentation could be attributed to the strain’s ability to produce new antioxidant compounds during fermentation through different metabolic pathways, or to their release from the cellular matrix. The progressive release of simple phenols by acid and the enzymatic hydrolysis of polyphenolic compounds could also contribute to this increase [42,48]. An increase in the antioxidant activity along fermentation has also been reported in other substrates, such as wolfberry juice [49] or carrot and beetroot juice [44]. The different trends observed in the antioxidant activity depending on the analytical method can be attributed to the different chemical principles involved. The ABTS assay quantifies both hydrophobic and hydrophilic compounds, while the DPPH method is more sensitive to hydrophobic compounds, usually resulting in lower values [50,51]. From a practical perspective, the use of more than one method provides a more comprehensive assessment of antioxidant capacity, since real food systems are usually a heterogeneous mixture of compounds with different polarities. Gao et al. [46] reported that total flavonoids content correlate significantly with DPPH and FRAP antioxidant methods, but no significant correlation was found between flavonoid content and ABTS antioxidant capacity, suggesting that factors other than total flavonoids may influence the results of the ABTS assay. Likewise, in the present work, total flavonoids exhibited an evolution over the course of fermentation, moving closer to DPPH antioxidant capacity than to ABTS, which showed an evolution pattern closer to the t.

### 3.2. Impact of Ultrasounds on the Physicochemical and Antioxidant Properties of Carrot Residues

Table 2 shows the values for the moisture content (x_w_), water activity (a_w_) and soluble solids content (x_ss_) of the carrot residues treated by ultrasounds (US) and the control (non-ultrasonicated).

Water content was not affected by US conditions, since no statistically significant differences were obtained for moisture content and only slight differences were observed for water activity, with no clear trend. In the case of soluble solids content (x_ss_), significantly higher values (*p*-value < 0.05) were obtained when 200 g of the sample was treated for 5 min (40 kHz). Other authors [52] found that glucose may be released from the side chains of hemicellulose with short US treatments, whereas longer treatments may lead to a decrease in soluble solids. In contrast, numerous studies on different vegetables claim that the longer the duration of ultrasound treatment, the higher the soluble solids content [53,54], which could be associated with the disruption of plant tissues and cell walls when ultrasonicated [55].

**Figure 3:**
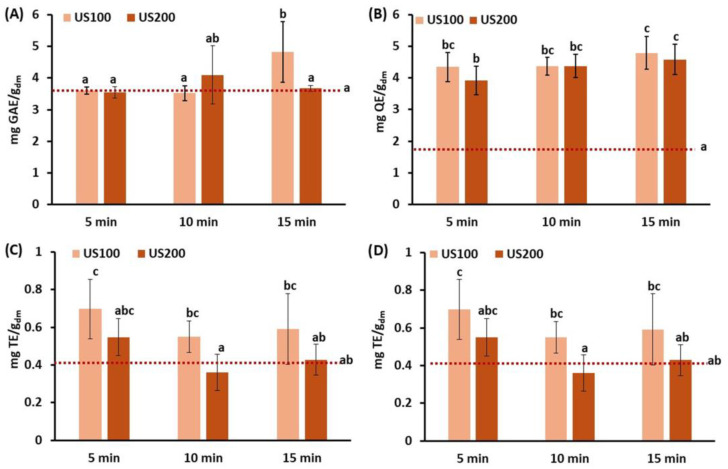
Antioxidant properties of sonicated carrot waste. (**A**) Total phenols content (mg GAE/g_dm_); (**B**) Total flavonoid content (mg QE/g_dm_); (**C**) DPPH antioxidant capacity (mg TE/g_dm_); (**D**) ABTS antioxidant capacity (mg TE/g_dm_). Discontinuous horizontal line represents the control sample (disrupted carrot waste). US100/200: ultrasonication at 40 kHz of a 100/200 g sample. Treatment duration: 5, 10, 15 min. Error bars represent the standard deviation of two independent samples (*n* = 2), measured in duplicate. ^a,b,c^: In each graph, different letters indicate statistically significant differences at the 95% confidence level.

The antioxidant properties of ultrasonicated carrot tissue as compared to the control are presented in Figure 3. Ultrasounds are expected to promote the disruption of the cellular structure and contribute to the release of antioxidant compounds, such as phenolic constituents. Shear stresses or microstreaming due to cavitation may contribute to antioxidant release, but other effects, such as bubble collapsing, may result in a temperature increase and degradation.

Among the different conditions studied, the sample that exhibited the highest total phenol content was 100 g ultrasonicated for 15 min, which resulted in a 1.3-fold increase as compared to the control. Other authors also reported better results when lengthening the US treatment [56,57,58,59]; a study on the effect of US treatment on the antioxidant properties of cape gooseberry showed an increase from 4.3% to 14.6% when the treatment was lengthened from 10 to 40 min [59]. It is evidenced that ultrasound-induced cavitation phenomena cause a mechanical rupture of the cell walls, facilitating the release of phenolic compounds naturally bound to the cell matrix. This would explain that longer treatments imply a greater degree of cell rupture, thus releasing more phenolic compounds that are bound to pectin, cellulose, hemicellulose and lignin [60,61]. Regarding the amount of sample that is processed, other studies also evidenced better results when reducing sample mass, which results in an energy density increase. For instance, a study on grapes that evaluated the ultrasonication of 0.5, 1 and 2 g samples found that the phenolic content was lower when ultrasonicating 2 g as compared to 1 g [62]. This result could be attributed to the fact that the energy available per unit of mass is higher in smaller samples at the same frequency and exposure time. In contrast, larger sample sizes may lead to reduced cavitation uniformity, causing areas of the sample to receive insufficient energy. Total flavonoid content (Figure 3B) showed a significant increase with US treatment, with samples exposed for 15 min standing out in both cases, reaching values 2.7 times higher than the untreated carrot residues. Similarly, in ultrasonicated grapefruit juice, the total flavonoid content increased when extending the treatment time up to 90 min [63].

In general, the treatment of carrot residue with US resulted in an increase in antioxidant capacity. According to the DPPH method, the antioxidant activity reached a maximum at 5 min of treatment, with values 1.3–1.7 times higher than those of the untreated carrot residue and subsequently decreased with treatment duration. A similar trend was observed in terebinth tree fruits when the treatment was extended from 30 to 45 min, which was attributed to the fact that the prolonged exposure of bioactive compounds to ultrasounds could led to their degradation [64]. However, when using the ABTS method, antioxidant capacity exhibited significant increases along treatment for all ultrasonicated samples as compared to the control (*p*-value < 0.05) (Figure 3D). In this case, although values increased with US time, no significant differences were found between 10 and 15 min, with ABTS scavenging activity reaching 1.4- and 1.6-fold higher than the control for US treatments applied to 100 g and 200 g of the sample, respectively.

### 3.3. Impact of Microwaves on the Physicochemical and Antioxidant Properties of Carrot Residues

This section evaluates the effect of microwave treatment (1.5 and 3 W/g) for 2, 4 and 6 min on the physicochemical and antioxidant properties of the carrot residue. Table 3 shows the values obtained for moisture content (x_w_), water activity (a_w_), and soluble solids content (x_ss_) in both microwave-treated carrot waste and control.

The moisture content of the carrot residue reached a maximum and minimum value after treatment at 3 W/g for 2 and 6 min, respectively. Changes in moisture content result from the relative balance between the simultaneous vaporization and the release of water from vacuoles during sample heating, resulting from the effect of microwaves on water molecules through dipolar polarization and ionic conduction [65]. When short treatments or low powers are applied, the release of water may predominate, leading to an increase in the measured moisture content. In contrast, lengthening the treatment and/or increasing power leads to a more pronounced evaporation, resulting in a reduction in moisture content. No statistically significant differences (*p*-value ≥ 0.05) were obtained for a_w_ values.

Low-power treatments (1.5 W/g) had little or even negative impact on soluble solids content (x_ss_), particularly when samples were microwaved for 2 min. This could be due to the reduced extractability associated with low-intensity treatments [17]. In contrast, the increase in soluble solids content when a higher power was applied (3 W/g) could be due to the enhanced extractability of soluble solids resulting from the disruption of plant matrix structures caused by microwaving [66]. As discussed in the work of Conesa et al. [17], fast evaporation promotes the disruption of cell walls and membranes at hot spots, where overheating occurs, compromising cells’ integrity and releasing the cellular contents.

**Figure 4:**
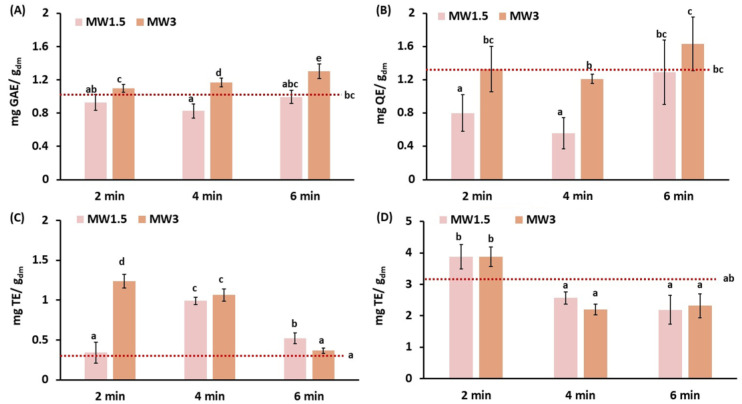
Antioxidant properties of microwaved carrot waste. (**A**) Total phenols content (mg GAE/g_dm_); (**B**) total flavonoid content (mg QE/g_dm_); (**C**) DPPH antioxidant capacity (mg TE/g_dm_); (**D**) ABTS antioxidant capacity (mg TE/g_dm_). The discontinuous horizontal line represents control sample (disrupted carrot waste). MW1.5: 1.5 W/g; MW3: 3 W/g. Duration of treatment: 2, 4 and 6 min. Error bars represent the standard deviation of two independent samples (*n* = 2), measured in duplicate. ^a,b,c,d^: Different letters for the same compound indicate statistically significant differences at a 95% confidence level.

The antioxidant properties of MW-treated samples and control are presented in Figure 4. Interaction with the electromagnetic field during microwaves treatment generates uneven heating, which may result in fast heating at localized hotspots. This phenomenon leads to the fast vaporization of trapped water, breaking the cellular matrix and releasing compounds included in cells and vacuoles or bound to complex carbohydrates [16,17,18]. After microwave treatments, total phenol content showed statistically significant differences (*p*-value < 0.05) among the different samples. The sample subjected to the highest MW power for a prolonged period (MW3_6) showed the highest increase in the total phenolic content, with a value 1.27 times higher than control. At this power (3 W/g), a progressive increase in phenolic content during the treatment was observed, in line with the results reported by Jokić et al. [67] on broccoli, and Conesa et al. [17] on pineapple waste intended for bioethanol production. This increase in phenolic contents might be attributed to the solubilization of lignin [17], but could also be due to the release of phenolic constituents bound to other cellular structures, particularly in biomasses that are not rich in lignin, such as broccoli. According to Pokkanta et al. [68], the phytochemicals in plant cell walls, such as phenolic compounds, may dissolve during microwave treatment due to the breakage of the bonds that connect them; other authors report an increase in the bioaccessibility of certain phenolic compounds due to microwave processing, particularly following microwave heating at 600 W for up to 30 min [69]. On the other hand, the lower MW powers and treatment duration (MW1.5_2 and MW1.5_4) implied a reduction in the phenolic content of samples. Excessive microwave exposure can degrade phytochemicals of natural products due to the electromagnetic force of microwaves, thermal accelerated oxidative deterioration and heat, especially in heat-sensitive substances such as polyphenols [68]. However, this might not be the reason for polyphenol depletion at low-intensity treatments. In contrast, lower solubilization or the reduced inhibition of oxidizing enzymes could be the reason for the reduction in phenolics under these treatment conditions.

In line with the total phenol content, total flavonoids exhibited the greatest increase in sample MW3_6, with a value 1.27 times higher than that of the control, whereas samples MW1.5_2 and MW1.5_4 experienced decreases of 38% and 57%, respectively. In a study by Jokić et al. on broccoli [67], the exposure time to microwaves was found to have a statistically significant effect on flavonoids content, with a proportional increase with power and exposure time. Zeb et al. [69] reported an initial decrease and a further increase in flavonoid content when increasing the duration of microwave treatment, suggesting that some flavonoids may degrade at low-intensity treatments and further be transformed into more reactive forms. The same authors observed variable behaviour in carotenoids’ and pigments’ response to microwave heating.

In the case of antioxidant activity measured by the DPPH radical method (Figure 4C), a considerable increase was observed with respect to the control sample in all conditions tested, except for low-intensity–short-time (MW1.5_2) and high-intensity–long-time (MW3_6). Increasing the microwave power during the shortest treatments led to an increase in antioxidant capacity. According to Quiles-Carrillo et al. [70], this effect may be attributed to the acceleration of intermolecular interactions, which enhances the solubility of different compounds. Nevertheless, MW are known to produce local explosions at hot spots due to uneven heating [17], which may result in the rupture of the cellular matrix, facilitating the release of compounds. Additionally, heating is also responsible for Maillard reactions and, eventually, the generation of melanoidins, which are large-stage reaction products with known antioxidant activity [71]. In fact, melanoidins are widely believed to occur during the hydrothermal pretreatment of organic solid waste [45].

The antioxidant activity measured by the ABTS radical method (Figure 4D) exhibited a different pattern. Short-duration treatment at both analyzed powers showed an ABTS radical scavenging ability close to that of the control, whereas increasing treatment duration had a negative impact, resulting in an average loss of about 31 ± 5%. This may be due to the degradation of certain antioxidant compounds due to high temperatures. During microwave heating, the generated heat acts on the polar bonds of the compounds, contributing to chemical reactions such as oxidation, dehydration, structural changes, and esterification, which can lead to reactions in secondary plant metabolites or their transformation into other structures [69]. The differences between the ABTS and DPPH methods were in line with those reported by Albarri and Şahin [50] for microwave-treated *Moringa oleifera* leaves.

### 3.4. Impact of Biological and Thermophysical Treatments on the Microstructure of Carrot Residues

**Figure 5:**
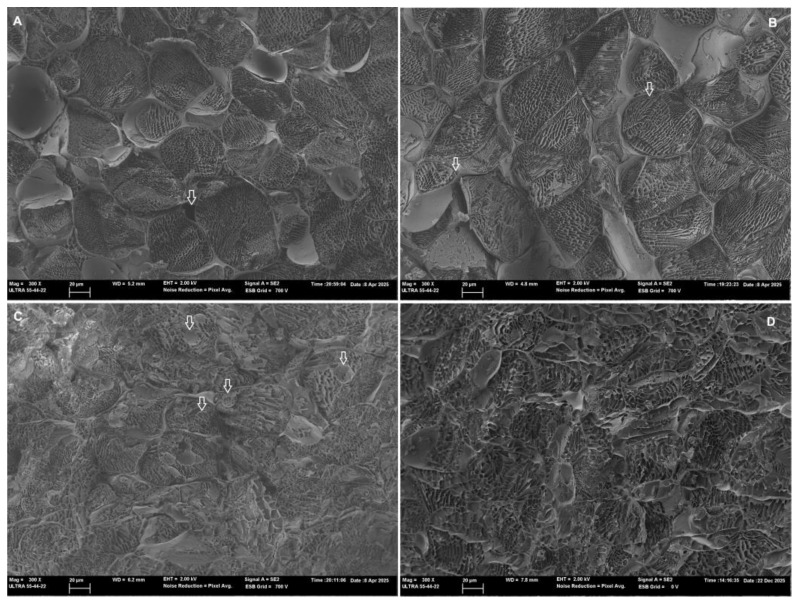
Cryo-FESEM micrographs of carrot residue tissue: Control (disrupted carrot waste) (**A**), ultrasonicated (**B**), microwaved (**C**) and fermented (**D**), observed at 300× of magnification (bar = 20 microns). White arrows indicate empty intercellular spaces in the fresh carrot tissue (**A**); signs of permeabilization in the ultrasonicated carrot tissue (**B**); and vesicles or globular plasma membrane structures in the microwaved sample (**C**).

Based on the observed improvement in the antioxidant properties of carrot residues, the following processing conditions were selected for microstructure evaluation: fermentation with *Ligilactobacillus salivarius* CECT 4063 for 24 h; ultrasonication of 100 g of the sample at 40 kHz for 15 min; and microwaving at 3 W/g for 4 min. The microstructural modifications of carrot residue produced by the application of selected treatments are presented in Figure 5.

Control samples (Figure 5A) exhibited classical parenchymatic tissue, with big, rounded cells in which the cell wall can be clearly identified. Intercellular spaces and pores can also be observed, some of them appearing empty (voids), a fact which suggests the preservation of tissue integrity [72]. Nevertheless, some intercellular spaces appear filled with liquid, which could be due to the release of part of the internal liquid phase during grinding. In the ultrasonicated samples (Figure 5B), cell integrity is also preserved; however, there are signs of membrane permeabilization due to cavitation phenomena, since membrane separation from the cell walls can be identified in several cells, likely due to mechanical vibration [73]. The slight structural changes evidenced in ultrasonicated samples through microscopic observation align with the improvement in the antioxidant properties observed when US is applied. In contrast, microwaved samples (Figure 5C) showed pronounced structural degradation. Accordingly, less defined cellular structures are observed, together with the formation of intracellular globular-like structures or aggregates. These changes were likely promoted by the fast evaporation of intracellular water during the local explosion phenomena due to the uneven heating characteristic of microwave treatment [24]. Located fast evaporation promotes the disruption of cell walls and membranes at hot spots, thus compromising cell integrity and releasing cellular contents [17]. As previously observed in broccoli tissues [24], the rounded structures that result from protoplast detachment and membrane invaginations due to fast dehydration were identified in microwaved carrot samples. These structures, which include sub-protoplasts, endocytic vesicles or globular-like structures, have also been observed in tissues subjected to osmotic treatments, or in response to salinity or freezing [74,75,76].

The impact of microwaves on the antioxidant properties of carrot wastes was heterogeneous according to the different trends observed in phenols and flavonoids, and the results of the antioxidant activity assays. While phenols and flavonoids showed a moderate increase with treatment intensification, the antioxidant activity decreased. The degradation of structures due to microwave treatment could have released some phenolic constituents bound to cellular structures, including flavonoids, but other antioxidant compounds could have been degraded due to the temperature increases that occur when intensifying treatment, particularly at hot spots. Regarding fermented samples (Figure 5D), micrographs revealed significant degradation of structures as compared to control and ultrasonicated tissue. This result is consistent with the action of LAB on the cellular matrix due to enzymatic and acid hydrolysis, as carrot wastes were used as the substrate in the fermentation process. The more remarkable increase in the antioxidant properties in fermented samples corresponded to total phenol content, suggesting that hydrolytic enzymes acted on cell structures, releasing previously bound phenolic molecules. However, only a slight improvement was observed in the antioxidant activity measurements, and only when the ABTS method was used. Overall, Cryo-FESEM observations suggested that moderate changes enhance bioaccessibility, whereas signs of more extensive damage coincide with the degradation of bioactive substances.

Changes in the cellular structure are known to have an impact on further processing, particularly at the dehydration stage [12]. Indeed, pronounced structural modifications are expected to reduce drying times and associated energy consumption, with the net effect on antioxidant properties depending on the balance between the release of bioactive compounds from the cell structure, the biochemical reactions taking place during drying, the limitations of temperature-sensitive oxidative enzymes, and the exposure to potentially adverse conditions, which may degrade the antioxidant compounds, depending on the dehydration technique used.

## 4. Conclusions

In this work, the impact of biological and thermophysical treatments on carrot wastes was evaluated. The three treatments applied, i.e., lactic acid fermentation, ultrasonication and microwave processing, effectively enhanced the antioxidant properties of fresh-cut carrot residues. However, their impact depended strongly on both the treatment conditions and the antioxidant parameter assessed.

In general terms, the differences among treatments can be explained by the distinct mechanisms characteristic of each particular treatment and their impact on the cellular structure and antioxidants’ release, and on the biochemical transformations induced by the treatments themselves. Based on total phenolic content, fermentation for 24 h with *Ligilactobacillus salivarius*, ultrasonication at 40 kHz applied to a 100 g sample for 15 min, and microwave treatment at 3 W/g for 6 min produced the greatest increases, with values in all cases being approximately 1.3-fold higher than the disrupted carrot tissue. These results were consistent with the release of bound phenolic compounds through microbial enzymatic activity during fermentation, cavitation-induced disruptions during ultrasonication, and extensive structural modifications promoted by the fast evaporation of intracellular water during microwave treatment, as supported by Cryo-FESEM microstructural observations. Other conditions, however, led to a decrease in the antioxidant properties of carrot residues. Their conversion to forms with different activity levels due to enzyme action or biochemical reactions may also have contributed to the increase in or depletion of antioxidant properties. Overall, ultrasonication provided the most balanced improvement in antioxidant properties. Nevertheless, obtaining the phenolic profile of the treated carrot waste using more precise techniques could help elucidate the changes in antioxidant compounds.

From an industrial perspective, all three treatments (ultrasonication, microwave, and fermentation) appear to be feasible for processing carrot residues and could reduce processing times if the wastes are further dried to produce functional powdered ingredients. Nevertheless, further studies are required to fully assess the scalability and consistency of these processes at the industrial level. To conclude, the findings of the present work highlight the need to optimize processing conditions to effectively valorize carrot residues. Future work will consider the combined effect of biological and thermophysical pretreatments with subsequent dehydration and milling stages, with the aim of developing functional powdered ingredients with an increased shelf life.

## Figures and Tables

**Table 1:** Moisture content (x_w_), water activity (a_w_), soluble solids content (x_ss_) and pH of fermented carrot residue during fermentation with *Ligilactobacillus salivarius CECT 4063*. Mean ± standard deviation of three independent samples (*n* = 3), measured in duplicate. Control: disrupted and pasteurized carrot residue; F_12, F_24 and F_48: carrot residue fermented with *L. salivarius* for 12, 24 and 48 h, respectively.

Sample	x_w_ (g_w_/100 g)	a_w_	x_ss_ (g_ss_/g_dm_)	pH
Control	87.89 ± 0.08 ^a^	0.993 ± 0.001 ^a^	0.998 ± 0.013 ^d^	5.512 ± 0.008 ^c^
F_12	89 ± 2 ^a^	0.9931 ± 0.0011 ^a^	0.834 ± 0.011 ^c^	3.83 ± 0.10 ^b^
F_24	88 ± 10 ^a^	0.990 ± 0.002 ^a^	0.78 ± 0.02 ^b^	3.63 ± 0.01 ^a^
F_48	91.2 ± 1.3 ^a^	0.994 ± 0.002 ^a^	0.76 ± 0.02 ^a^	3.68 ± 0.12 ^a^

^a–d^ different letters in the same column indicate statistically significant differences at the 95% confidence level.

**Table 2:** Physicochemical properties of ultrasonicated carrot residues. Mean ± standard deviation of three independent samples (*n* = 2), measured in duplicate. Control: disrupted carrot waste. US, ultrasonicated; 100/200, amount of sample (g); 5/10/15, exposure time (min).

Sample	x_w_ (g_w_/100 g)	a_w_	x_ss_ (g_ss_/g_dm_)
Control	91.3 ± 0.4 ^a^	0.9941 ± 0.0004 ^b^	0.755 ± 0.007 ^a^
US100_5	90.9 ± 0.2 ^a^	0.9890 ± 0.0013 ^a^	0.767 ± 0.007 ^b^
US100_10	91.4 ± 0.2 ^a^	0.995 ± 0.003 ^b^	0.759 ± 0.012 ^a^
US100_15	91.12 ± 0.11 ^a^	0.9942 ± 0.0008 ^b^	0.754 ± 0.007 ^a^
US200_5	91.5 ± 1.7 ^a^	0.999 ± 0.003 ^c^	0.785 ± 0.014 ^c^
US200_10	91.0 ± 0.2 ^a^	0.995 ± 0.003 ^b^	0.771 ± 0.012 ^bc^
US200_15	91.4 ± 0.4 ^a^	0.996 ± 0.002 ^bc^	0.767 ± 0.007 ^b^

^a,b,c^ different letters in the same column indicate statistically significant differences at the 95% confidence level.

**Table 3:** Physicochemical properties of microwave-treated carrot residue. Mean ± standard deviation of three independent samples (*n* = 2), measured in duplicate. MW1.5 and MW3 correspond to 1.5 and 3 W/g, whereas 2, 4, 6 refer to the exposure time. Control: disrupted carrot waste.

Sample	x_w_ (g_w_/100 g)	a_w_	x_ss_ (g_ss_/g_dm_)
Control	90.313 ± 0.008 ^b^	0.994 ± 0.003 ^a^	0.727 ± 0.009 ^b^
MW1.5_2	90.3 ± 0.2 ^b^	0.996 ± 0.002 ^a^	0.66 ± 0.03 ^a^
MW1.5_4	90.08 ± 0.12 ^b^	0.9938 ± 0.0002 ^a^	0.727 ± 0.007 ^b^
MW1.5_6	90.0 ± 0.3 ^b^	0.9924 ± 0.0011 ^a^	0.705 ± 0.012 ^ab^
MW3_2	92.1 ± 1.9 ^c^	0.993 ± 0.002 ^a^	0.74 ± 0.03 ^b^
MW3_4	89.3 ± 0.4 ^b^	0.994 ± 0.002 ^a^	0.816 ± 0.007 ^c^
MW3_6	87.10 ± 0.12 ^a^	0.9925 ± 0.0013 ^a^	0.92 ± 0.07 ^d^

^a to d^: Different letters in the same column indicate statistically significant differences at the 95% confidence level.

## Data Availability

The data supporting the findings of this study are available from the corresponding author upon reasonable request.
